# Protocol-directed insulin infusion sliding scales improve perioperative hyperglycaemia in critical care

**DOI:** 10.1186/2047-0525-1-7

**Published:** 2012-10-06

**Authors:** Man Lin Hui, Arun Kumar, Gary G Adams

**Affiliations:** 1 The Queen Elizabeth Hospital, 30 Gascoigne Road, Kowloon, Hong Kong; 2 Faculty of Medicine and Health Science, University of Nottingham, Clifton Boulevard, Nottingham, NG7 2RD, UK; 3 Insulin and Diabetes Experimental Research (IDER) Group, Faculty of Medicine and Health Science, University of Nottingham, Clifton Boulevard, Nottingham, NG7 2RD, UK

**Keywords:** Hyperglycaemia, Perioperative, Protocol-directed insulin infusion, Sliding scales

## Abstract

Perioperative hyperglycaemia is associated with poor outcomes in patients undergoing cardiac surgery. Frequent postoperative hyperglycaemia in cardiac surgery patients has led to the initiation of an insulin infusion sliding scale for quality improvement.

A systematic review was conducted to determine whether a protocol-directed insulin infusion sliding scale is as safe and effective as a conventional practitioner-directed insulin infusion sliding scale, within target blood glucose ranges.

A literature survey was conducted to identify reports on the effectiveness and safety of an insulin infusion protocol, using seven electronic databases from 2000 to 2012: MEDLINE, CINAHL, EMBASE, the Cochrane Library, the Joanna Briggs Institute Library and SIGLE. Data were extracted using pre-determined systematic review and meta-analysis criteria.

Seven research studies met the inclusion criteria. There was an improvement in overall glycaemic control in five of these studies. The implementation of protocols led to the achievement of blood glucose concentration targets more rapidly and the maintenance of a specified target blood glucose range for a longer time, without any increased frequency of hyperglycaemia. Of the seven studies, four used controls and three had no controls.

In terms of the meta-analysis carried out, four studies revealed a failure of patients reaching target blood glucose levels (*P* < 0.0005) in the control group compared with patients in the protocol group. The risk of hypoglycaemia was significantly reduced (*P* <0.00001) between studies.

It can be concluded that the protocol-directed insulin infusion sliding scale is safe and improves blood glucose control when compared with the conventional practitioner-directed insulin infusion sliding scale. This study supports the adoption of a protocol-directed insulin infusion sliding scale as a standard of care for post-cardiac surgery patients.

## Review

### Introduction

Hyperglycaemia is a problem associated with blood glucose levels in excess of 10 mmol/l; it is a common occurrence in cardiac surgery patients and is associated with adverse outcomes [1]. Prolonged hyperglycaemia increases the risk of infection and contributes to higher mortality and morbidity. Mounting evidence documents the beneficial effects of tight glycaemic control on patients’ recoveries [2] and highlights the importance of avoiding hyperglycaemic-related complications in coronary artery bypass graft patients, for effective postoperative glycaemic control.

Temporary hyperglycaemia during stress is often helpful and helps to provide more glucose to prepare the individual for action [3]. However, hyperglycaemia in a critically ill [4] or postoperative patient [5] may have various detrimental effects on the host’s defence system: blood glucose levels >180 mg/dl (10 mmol/l) have a compromising effect on the immune system [6]; the immune responsiveness of the mononuclear phagocytic cells is depressed; neutrophil function is impaired; the inflammatory response is exaggerated; and the immune system is weakened, thus increasing susceptibility to infection [7]. Recent evidence has proved that perioperative (intraoperative plus postoperative) hyperglycaemia is directly correlated with the development of deep sternal wound infection, increased mortality and morbidity, and increased hospital stay [8]. Furnary [6] reported that the rate of wound infection was doubled when blood glucose levels were between 180 mg/dl (10 mmol/l) and 216 mg/dl (12 mmol/l), fourfold when they were between 216 mg/dl (12 mmol/l) and 252 mg/dl (14 mmol/l) and even sixfold when over 252 mg/dl (14 mmol/l); when blood glucose levels were maintained at below 180 mg/dl (10 mmol/l), there was no increase in the rate of wound infection [6].

Treating hyperglycaemia in hospitalized patients has proven to be beneficial [9]. However, normoglycaemia after cardiac surgery is usually difficult to maintain and requirements for insulin after cardiac surgery with cardiopulmonary bypass are much higher than after other operations.

Because the use of the cardiopulmonary bypass machine necessitates the administration of catecholamines and corticosteroids during and after cardiac surgery, the patient’s insulin resistance status changes continuously, thus altering the patient’s insulin response and causing fluctuations in glycaemia.

Recognizing the detrimental impact of hyperglycaemia on postoperative surgical wound infection and the importance of glycaemic control in cardiac surgery patients is important and appropriate in managing hyperglycaemic control. To address this issue, a systematic review was carried out to examine the effectiveness and safety of the protocol-directed insulin infusion sliding scale variable rate intravenous insulin infusion on this population group.

### Design of the study

The electronic databases MEDLINE and CINAHL were searched to identify keywords and index terms used in describing relevant studies. Keywords used in the preliminary searching process included: ‘insulin infusion sliding scale’, ‘open-heart surgery’, ‘practitioner-directed’ and ‘protocol-directed’.

**Figure 1:**
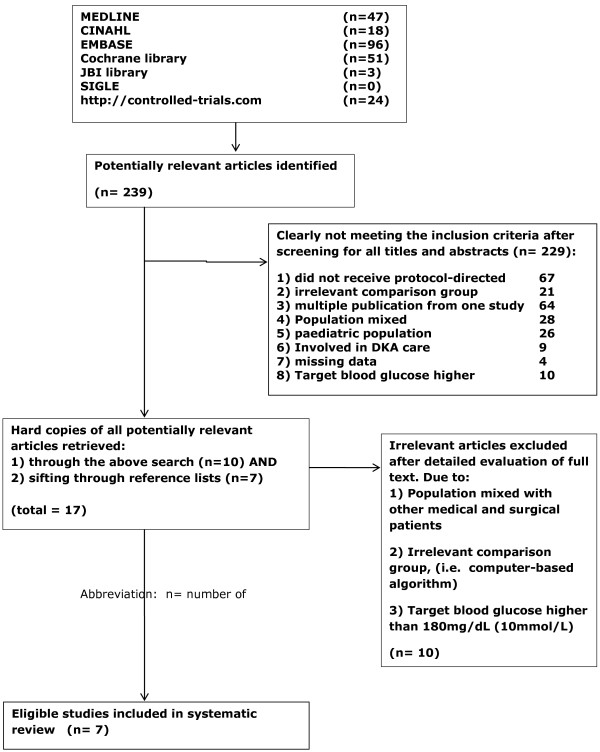
**Flowchart of study selection.** The data in this figure refer to the original search, completed in 2011.

A more detailed and extensive search was then conducted across a number of electronic databases to ensure that the majority of studies within the inclusion criteria were recruited (Figure 1). Databases that cover the healthcare literature and clinical trials were searched. To increase the coverage of all relevant evidence, different databases were used in the searching process: MEDLINE, CINAHL, the Cochrane Library, the Joanna Briggs Institute (JBI) Library, and EMBASE. To identify published studies that are not available electronically, hand searching was also done. In addition, as it can take more than a year for some studies to be published, and these studies may not be searchable in electronic databases, a manual journal search was also performed.

Unpublished studies were sought, to overcome or reduce publication bias, using the System of Information on Grey Literature in Europe (SIGLE) database.

The bibliographies and reference lists from the recruited articles were consulted to identify additional studies for possible inclusion in this review.

The outcomes of interest are the effectiveness and safety of the insulin infusion sliding scale in controlling blood glucose level. The efficiency of an insulin infusion sliding scale in controlling blood glucose levels and reducing them to within the normal range plays a large role in dictating the scale’s use in clinical practice. So efficiency was set as one of the outcome of interest. In addition, any insulin infusion sliding scale used to lower the blood glucose level may induce hypoglycaemia, which can lead to devastating effects, such as irreversible neurologic deficit [10]. Therefore, safety of an insulin infusion scale is another important factor that needs to be taken into consideration.

To prevent publication bias, all published and unpublished studies that were written in English and met the inclusion criteria were included. In addition, to identify only the most up-to-date studies, only those published after 2000 were included.

### Type of participant

All adult patients, over 18 years old who had undergone open-heart surgery with blood glucose level >180 mg/dl (10 mmol/l) and needed insulin therapy, with or without comorbidities will be eligible for inclusion, except those patients who developed diabetes ketoacidosis.

Of the 239 potentially relevant articles identified during the primary search, and after screening all titles and abstracts, 229 clearly did not meet the inclusion criteria and were therefore excluded. Hard copies of all potentially relevant articles were retrieved, including those obtained directly from the search (*n* = 10) and those obtained through reference lists (*n* = 7). Irrelevant articles were excluded after detailed evaluation of the full text (Figure 1).

There was usually more than one reason for excluding each study from the analysis; these included:

1) Not all participating patients received protocol-directed blood glucose management (67 articles).

2) There was an irrelevant comparison group, (for example, a computer-based algorithm was used instead of a control group) (21 articles).

3) Data from one study were used in more than one publication (in the form of quality of life data) (64 articles).

4) The population studied included other medical and surgical patients (28 articles).

5) The report studied a paediatric population (26 articles).

6) The patients studied were receiving diabetes ketoacidosis care (9 articles).

7) There were missing data for patients receiving study medication (4 articles).

8) The target blood glucose was higher than 180 mg/dl (10 mmol/l) (10 articles).

This left seven studies eligible for systematic review (Table 1).

**Table 1:** Protocol-directed insulin infusion improve perioperative hyperglycaemia in critical care

**Reference**	**Number of patients**	**Method**	**Target blood glucose concentration**	**Frequency of measurement**	**Frequency of hyperglycaemia**	**Main results**
Zimmerman *et al*. (2004) 16	168 postoperative cardiothoracic surgical intensive care patients	A nurse-driven insulin infusion protocol was developed and implemented in postoperative cardiothoracic surgical intensive care patients with or without diabetes.	80 to 150 mg/dl (4.4 to 8.3 mmol/l)	Every 1 to 4 hours	12 patients (7.1%) <40 mg/dl (2.2 mmol/l)	Findings showed percentage and time of blood glucose measurements within the tight glycaemic control range (control 47% vs. protocol 61%; *P* = 0.001),
		This before-and-after cohort study used two periods of measurement: a 6-month baseline period prior to the initiation of the insulin			28 patients (16.7%) <65 mg/dl (3.6 mmol/l)	Area under curve (AUC) of glucose exposure >150 mg/dl (8.3mmol/l) vs. time for the first 24 hours of the insulin infusion (control 28.4 vs. protocol 14.8; *P* < 0.001), median time to blood glucose <150 mg/dl (8.3mmol/l) (control 9.4 h vs. protocol 2.1h; *P* < 0.001), and percentage blood glucose <65 mg/dl (3.6 mmol/l) as a marker for hypoglycaemia (control 9.8% vs. protocol 16.7%; NS).
		infusion protocol (control group, *n* = 174) followed by a 6-month intervention period, in which the protocol was used (protocol group, *n* = 168).				
Tamaki *et al*. (2008) 17	40 cardiac surgery patients	The Yale insulin infusion protocol was modified by taking into consideration the characteristics of Japanese diabetics and the hospital environment.	80 to 140 mg/dl (4.4 to 7.8 mmol/l)	Every 30 min to every 2 hours	Blood glucose values <60 mg/dl (3.3mmol/l) 0.5% ± 5.9%	Analyses of 1,656 blood glucose measurements during insulin infusion revealed that the percentage of samples that showed achievement of target blood glucose level (80 to 140 mg/dl (4.4 to 7.8 mmol/l)) was higher under protocol (78 ± 15%, *n* = 870) than control (57 ± 23%, *n* = 786, *P* < 0.0001).
		The modified protocol was tested in 40 type-2 diabetic patients after elective open-heart surgery, compared with 35 type-2 diabetic patients under empirical blood glucose control.				On the other hand, the fraction of samples with blood glucose <60 mg/dl (3.3 mmol/l) was comparable in the two groups (protocol: 0.5 ± 5.9‰, control: 5.1 ± 18.5‰).
						None of the patients with hypoglycaemia showed significant clinical adverse effects.
Caddell *et al*. (2010) 18	100 cardiovascular surgery patients	Prospective data were gathered on 100 consecutive cardiovascular surgery patients managed with standard insulin infusion protocol and 100 patients managed with an insulin-resistance-guided protocol. Clinical characteristics and glycaemic indices were analyzed for the two groups. Primary outcomes included: percentage of time spent in the target range; number of hypoglycaemic and hyperglycaemic episodes; time to achievement of target blood glucose concentration; and total daily dose of insulin required.	80 to 110 mg/dl (4.4 to 6.1 mmol/l)	Hourly	<70 mg/dl (3.9 mmol/l): 0.12 event per patient	The insulin-resistance guided protocol resulted in significant improvements, including increased percentage of time spent in the normoglycaemic range (82.5% vs. 65.8%, *P* < 0.001), reduced rate of hypoglycaemic episodes (0.12 vs. 0.99, *P* < 0.01), reduced rate of hyperglycaemic episodes (capillary blood glucose >126 mg/dl (7 mmol/l): 4.8 vs. 8.2, *P* < 0.01), and reduced time to the first measurement in the target range. Total daily dose of insulin was mildly increased, but failed to reach statistical significance (92.48 vs. 82.64 units, *P* = 0.32).
					<40 mg/dl (2.2 mmol/l): 0.04 event per patient	
Leibowitz *et al*. (2010) 31	203 cardiac surgery patients	Patients with diabetes mellitus or random blood glucose >150 mg/dl were treated in the intensive care unit with intravenous insulin, followed by a multi-injection protocol consisting of four glargine-aspart insulin injections in the ward, with a glycaemic target of 110 to 150 mg/dl (6.1 to 8.3 mml/l).	110 to 150 mg/dl (6.1 to 8.3 mmol/l)	Every 20 min to every 4 hours	3% patients with blood glucose <60mg/dl (3.3 mmol/l)	During the intervention, mean blood glucose ± SD was 151 ± 19 mg/dl (8.4 ± 1.1 mmol/l) and 157 ± 32 mg/dl (8.7 ± 1.8 mmol/l) in the intensive care unit and ward, respectively, vs. 166 ± 27 mg/dl (9.2 ± 1.5 mmol/l) and 184 ± 46 mg/dl (10.2 ± 2.6 mmol/l) during the control period (*P* < 0.0001). The incidence of hypoglycaemia (blood glucose less than 60 mg/dl) was low and similar in the two groups (2.5% control vs. 3% intervention). Intensive insulin treatment decreased the risk for infection from 11% to 5% (56% risk reduction, *P* = 0.018), mainly by reducing the incidence of graft harvest site infection (6.9% vs. 2.5%, *P* = 0.034). The incidence of atrial fibrillation after coronary artery bypass graft surgery decreased from 30% to 18% (39% risk reduction; *P* = 0.042).
		The study cohort (*n* = 410) consisted of consecutive patients undergoing cardiothoracic surgery. Control patients (*n* = 207) were admitted during the first 8 months				
		(***CONTROL GROUP)***				
		The intervention group of patients (*n* = 203) were operated on during the following 8 months.				
		The main outcome measures were glycaemic control and the rate of postsurgery infection.				
Goldberg *et al*. (2004) 24	118 cardiothoracic intensive care unit patients	A standardized, intensive insulin infusion protocol was used for all patients admitted to two cardiothoracic intensive care unit s. Hourly blood glucose levels, relevant baseline variables, and clinical interventions were collected prospectively from the active hospital chart and cardiothoracic intensive care unit nursing records.	100 to 139 mg/dl (5.6 to 7.7 mmol/l)	Hourly	Five blood glucose values (0.2%) <60 mg/dl (3.3 mmol/l)	The insulin infusion protocol was used 137 times in 118 patients. The median time required to reach target blood glucose levels (100 to 139 mg/dl (5.6 to 7.7 mmol/l)) was 5 hours. Once blood glucose levels decreased below 140 mg/dl, 58% of 2242 subsequent hourly blood glucose values fell within the target range, 73% within a ‘clinically desirable’ range of 80 to 199 mg/dl (4.4 to 11 mmol/l). Only five (0.2%) blood glucose values were less than 60 mg/dl (3.3 mmol/l), with no associated adverse clinical events.
					Lowest recorded blood glucose value: 48 mg/dl (2.7 mmol/l)	
Lecomte *et al.* (2008) 25	651 cardiac surgery patients	483 nondiabetics and 168 diabetics scheduled for cardiac surgery with cardiopulmonary bypass were recruited.	80 to 110 mg/dl (4.4 to 6.1 mmol/l)	Every 30 min to every 2 hours	Blood glucose values <60mg/dl (3.3 mmol/l)	18,893 blood glucose measurements were made during and after surgery. During surgery, the mean glucose level in nondiabetic patients was within targeted levels except during (112 ± 17 mg/dl (6.2 ± 0.9mmol/l)) and after rewarming (113 ± 19 mg/dl (6.3 ± 1.1mmol/l)) on cardiopulmonary bypass.
		To anticipate rapid perioperative changes in insulin requirement or sensitivity during surgery, a dynamic algorithm presented in tabular form, with rows representing blood glucose ranges and columns representing insulin dosages based on the patients’ insulin sensitivity was developed. The algorithm adjusts insulin dosage based on blood glucose level and the projected insulin sensitivity (for example, reduced sensitivity during cardiopulmonary bypass and normalizing sensitivity after surgery).			In nondiabetic patients: 0.16%	In diabetics, blood glucose was decreased from 121 ± 40 mg/dl (6.7 ± 2.2 mmol/l) at anaesthesia induction to 112 ± 26 mg/dl (6.2 ± 1.4 mmol/l) at the end of surgery (*P* < 0.05), with 52.9% of patients achieving the target.
					In diabetic patients: 0.22%	In the intensive care unit, the mean glucose level was within the targeted range at all times, except for diabetics on arrival at the intensive care unit (113 ± 24 mg/dl (6.3 ± 1.3mmol/l)).
					Lowest recorded blood glucose value: 40 mg/dl	Of all blood glucose measurements (operating room and intensive care unit), 68.0% were within the target, with 0.12% of measurements in nondiabetics and 0.18% in diabetics below 60 mg/dl (3.3 mmol/l). Hypoglycaemia <50 mg/dl (2.8mmol/l) was avoided in all but four (0.6%) patients (40 mg/dl (2.2mmol/l) was the lowest observed value).
					(2.2mmol/l)	
Studer *et al*. (2010) 26	230 cardiac surgery patients	230 consecutive patients (mean ± SD age: 67 ± 11 years; diabetic patients: *n* = 62) undergoing cardiac surgery (coronary artery bypass grafting: *n* = 137; 20% off-pump) or intrathoracic aortic (*n* = 10) surgery were included.	100 to 139 mg/dl (5.6 to 7.7 mmol/l)	Every 1 to 3 hours	Blood glucose <75 mg/dl (4.2 mmol/l): Postoperative day 1: 12 patients (5.3%)	All patients received postoperative insulin therapy. Patients spent 57.3% and 69.7% of time within the blood glucose target range on postoperative days 1 and 2, respectively. The percentage of time was significantly higher in nondiabetics than in diabetics. Mean blood glucose measurements per patient intraoperatively, on postoperative days 1 and 2 were 4 ± 1, 10 ± 2 and 7 ± 2, respectively. No patient experienced any severe hypoglycaemic events (blood glucose <50 mg/dl (2.8mmol/l)).
		Blood glucose control was managed according to an insulin therapy protocol, described by Goldberg *et al*. [24], in use for 6 months. Insulin infusion rate and frequency of blood glucose monitoring were adjusted according to: (1) the current blood glucose			Postoperative day 2: 7 patients (3.1%).	
		value; (2) the previous blood glucose value; and (3) the current insulin infusion rate. Efficacy was assessed by the percentage of time spent at the target blood glucose level (100 to 139 mg/dl (5.6 to 7.7mmol/l)) intraoperatively and during the first two postoperative days.			Blood glucose <50 mg/dl) (2.78%): 0 patients (0%)	

### Description of interventions

Interventions of interest will be limited to the use of the protocol-directed insulin infusion sliding scale; (2) a newly developed insulin infusion protocol; or a protocol modified from an existing protocol. For comparison, a practitioner-directed insulin infusion sliding scale and a conventional simple insulin infusion sliding scale will be included. Studies examining other types of insulin infusion sliding scale (such as computer-directed scales) will be excluded.

### Type of analysis

The objective of this study was to conduct a systematic review to determine whether a protocol-directed insulin infusion sliding scale is as safe and effective as the practitioner-directed insulin infusion sliding scale in bringing blood glucose values within the target range in a practical and real-life setting.

To minimize any errors and subjective judgement in the decision process, a critical appraisal instrument developed from the Joanna Briggs Institute (JBI-MAStARI) was used to appraise the methodological validity and quality of the studies independently [11]. This checklist required the authors to rate each individual study into one of three levels of credibility [12].

Data extraction was carried out by two reviewers independently to minimize errors. Data were extracted and stored using the standardized data extraction tool developed by JBI-MAStARI. Inconsistency in extracted data was settled by discussion between two reviewers. Where an agreement could not be reached, the problem was referred to a third reviewer for a decision.

Meta-analysis was carried out where appropriate and pooled using meta-analytical methods within Review Manager (RevMan) Version 5.1 software. No ethical approval was needed for this study.

Permission was obtained to use the databases and data within this study.

No ethical approval was required.

### Results and discussion

Perioperative hyperglycaemia has been shown to be associated with adverse surgical outcomes in cardiac surgery patients [13,14]. Effective hyperglycaemic treatment is, therefore, of significant benefit in all patients after cardiac surgery [15]. Here, we present the results of a number of studies where protocol-directed insulin infusions improve perioperative hyperglycaemia in critical care. These are divided into those with and without the use of controls in their studies.

The studies carried out by Zimmerman *et al*. [16], Tamaki *et al*. [17], Caddell *et al*. [18] and Leibowitz *et al*. [19] all showed positive correlations between the ability to attain target blood glucose levels and the infusion regimen used when compared with the controls.

This is exemplified in the study of Zimmerman *et al*. [16], who examined 168 (protocol group) and 174 (control group) patients in the cardiothoracic intensive care unit. The results clearly showed that the target blood glucose range was achieved within 2.1 hours of treatment compared with those patients on the standard sliding scale, where it took 9.4 hours to achieve the target blood glucose range, a significance level of *P* < 0.001. Moreover, 61% of all blood glucose measurements were within the target range (protocol group) compared with 47% (control group). Furthermore, the protocol group remained within the target range for longer (65.4%) compared with the control group (54.6%).

The glycaemic level in the protocol group may be lower than observed, owing to the administration of corticosteroids and their ability to aggravate hyperglycaemic status [20]. Nevertheless, the results demonstrated that implementation of the protocol led to a significant improvement in glycaemic control. The speed with which this control was achieved, however, is controversial because this study was associated with the highest level of hypoglycaemia among all seven studies. This indicates that too-rapid lowering of blood glucose levels could be dangerous in cardiac diabetic surgery patients, which is supported by a randomized controlled trial of 10,251 patients that demonstrated that rapidly lowering blood glucose concentrations in patients with type 2 diabetes might harm patients by precipitating hypoglycaemia [21]. The safety of this particular protocol is, however, called into question when 16.7% of patients in the protocol group were subjected to levels of <65 mg/dl (3.6 mmol/l) or less compared with 9.8% of patients in the control group.

In another protocol versus control study, Tamaki and co-workers evaluated the effectiveness and safety of a modified Yale insulin infusion protocol [17], to maintain blood glucose levels at 80 to 140 mg/dl (4.4 to 7.8 mmol/l) in 40 Japanese diabetic patients who had undergone cardiac surgery. The rate of change of insulin infusion was modified to ensure effective and safe use for Asian patients [22].

Once again, there was a positive correlation between the treatments used in the protocol group compared with those used in the control group. The patients in the protocol group reached their target blood glucose levels more quickly (3.1 ± 2.1 hours) compared with the control group (5.0 ± 3.3 hours). In addition, of the total 870 blood glucose measurements, 78 ± 15% were within the target range (protocol group) compared with 57 ± 23% of the total 786 measurements in the control group (*P* <0.0001).

Although this supports the work carried out by Zimmerman *et al*. [16], Tamaki’s work is still the only study carried out on Asian cardiac surgery patients, and it is difficult to correlate these results directly with those carried out in countries that utilize alternative protocols. Moreover, the small sample size of this study limited the generalizability of this particular study and the ability to capture the true clinical impact on this population.

Caddell *et al*. [18] introduced an insulin-resistance guided protocol, which demonstrated similar findings to those obtained in the Zimmerman and Tamaki studies. Caddell’s group showed that the insulin-resistance guided protocol led to more rapid and effective blood glucose control in cardiac surgery patients with target blood glucose levels of 80 to 110 mg/dl (4.4–6.1 mmol/l) in the first 24 hour postoperative period.

In the protocol group, 65% of patients reached target blood glucose ranges within 3 hours compared with 65% of the patients (control group), who reached the target range within 9 hours, (*P* < 0.01). Additionally, patients in the protocol group maintained their blood glucose concentration within the target range longer (82.5% (19.8 hours)) than the control group (65.8% (15.7 hours)), a significance level of *P* < 0.001.

The principal strength of Leibowitz’s study [19] over the Zimmerman, Tamaki and Caddell studies is the comparatively large number of patients. The study cohort consisted of 410 patients undergoing cardiothoracic surgery. The control patients (*n* = 207) were admitted during the first 8 months, whereas the intervention group of patients (*n* = 203) were operated on during the following 8 months.

The percentage of patients maintaining a target blood glucose level of 110 to 150 mg/dl (6.1 to 8.3 mmol/l) was 55% (protocol group) and 39% (control group) (*P* < 0.0001), although the effective achievement of target blood glucose control resulted in a small increase in the frequency of hypoglycaemia (3% vs. 2.5%), which was not considered significant. Nevertheless, the frequency of hypoglycaemia was still lower than that observed in other studies, which used the same target blood glucose range (110 to 150 mg/dl (6.1 to 8.3 mmol/l)) and was associated with 5% of hypoglycaemic events in cardiac care unit patients [23].

Three studies that were carried out without the use of controls were those of Goldberg *et al*. [24], Lecomte *et al*. [25] and Studer *et al*. [26]. Although no controls were used, insulin infusion protocols were used to obtain target blood glucose levels of 100 to 139 mg/dl (5.6 to 7.7 mmol/l), 80 to 110 mg/dl (4.4 to 6.1 mmol/l) and 100 to 139 mg/dl (5.6 to 7.7 mmol/l), respectively.

Goldberg’s group [24] investigated the use of an insulin infusion protocol in 118 patients (protocol group). The median time required to achieve the target glycaemic level was 5 hours. When blood glucose levels fell below 140 mg/dl (7.8 mmol/l), 58% of 2242 subsequent hourly blood glucose values fell within the target range. The strength and effectiveness of Lecomte’s study [25] stemmed from the large number of patients (651 patients) included, in addition to the homogeneity of the sample population. Results showed that the protocol achieved the target blood glucose level faster than Goldberg’s, in 3 hours (72.4% = nondiabetic) and (66.2% = diabetic patients). Studer’s study [26] demonstrated that under treatment conditions, nondiabetic patients achieved a better glycaemic control with a lower incidence of hypoglycaemic events than diabetic patients and is consistent with a previous study [27]. There were only four observed cases of hypoglycaemia in Lecomte’s study [25] and there were no associated adverse clinical episodes in the work carried out by Goldberg’s group [24].

The effectiveness and safety of these protocols rests with the fact that clinicians have pre-existing knowledge of previous blood glucose values, current blood glucose values and current insulin infusion rates, and this is confirmed by the meta-analysis of the four studies with controls [16-19], which showed that the percentage failure of patients reaching target blood glucose levels demonstrated a significant difference (*P* < 0.0005) from patients failing to achieve target blood glucose levels in the control group compared with patients treated by protocol (Additional file 1: Table S2).

Owing to the stress of surgery and the use of catecholamine and steroids during the perioperative period, patient’s blood glucose values can fluctuate immediately postoperatively [28]. Therefore, a dynamic protocol that regulates insulin dosage according to the relative change of blood glucose concentration, rather than one absolute blood glucose value is of great important in achieving tight glycaemic control effectively without increasing the risk of hypoglycaemia.

Intensive insulin management [29] is often required to optimize glycaemic control but this can be associated with insulin mismanagement, and severe hypoglycaemia is possible [30]. Hypoglycaemia, which requires emergency medical assistance, is commonplace in patients with longstanding insulin-treated type 1 and type 2 diabetes. Left untreated, severe hypoglycaemia can result in morbidity and death. Severe hypoglycaemia [31] can be prevented by utilizing appropriate medications and medication regimens [32], and effective glucose monitoring strategies [33] and technologies [34]. This is fully supported by our meta-analysis on the risk of hypoglycaemia on the studies and the subgroup (Additional file 2: Table S3) examined, in which the risk of hypoglycaemia was significantly reduced compared with control (*P* < 0.00001) between studies that used an insulin infusion protocol.

## Conclusion

Perioperative hyperglycaemia is associated with poor outcomes in patients undergoing cardiac surgery. Frequent postoperative hyperglycaemia in cardiac surgery patients has led to the instigation of a quality improvement insulin infusion sliding scale. A systematic review was conducted, to determine whether a protocol-directed insulin infusion sliding scale was as safe and effective as conventional practitioner-directed insulin infusion sliding scales. Seven research studies met the inclusion criteria. Five studies compared their insulin infusion protocols to the previous blood glucose management practice. Overall glycaemic control showed an improvement in all five studies. Of the seven studies, four used controls and three had no controls. Implementation of protocols led to blood glucose concentrations being achieved more readily. Moreover, blood glucose ranges were maintained for a longer time, without any increased frequency of hyperglycaemia.

### Key messages

The protocol-directed insulin infusion sliding scale is a safe and effective method.

Blood glucose control is improved when compared with the conventional practitioner-directed insulin infusion sliding scale.

This study supports the adoption of a protocol-directed insulin infusion sliding scale as a standard of care for post-cardiac surgery patients.

An effective protocol should be based on the velocity of glycaemic changes and patient’s insulin sensitivity.

The current blood glucose level, previous blood glucose levels and relative change of blood glucose levels between two consecutive measurements, as well as the patient’s insulin resistance status, are clinically important and should be used as parameters of care, instead of relying solely on the latest blood glucose level itself to adjust insulin infusion rates.

## Abbreviations

JBI: Joanna Briggs Institute; SIGLE: Open System for Information on Grey literature in Europe.

## Competing interests

The authors have no competing interests.

## Authors’ contributions

Conception and design: MLH/GGA; Analysis and interpretation: MLH/GGA; Drafting the manuscript for important intellectual content:MLH/AK/GGA. All authors read and approved the final manuscript.

## Supplementary Material

Additional file 1 **Table S2.** Percentage failure to reach target blood glucose levels. Click here for file

Additional file 2 **Table S3.** Risk of hypoglycaemia. Click here for file
