# Supplementary material for: Protocol-directed insulin infusion sliding scales improve perioperative hyperglycaemia in critical care
Source: Perioper Med (Lond). 2012 Oct 6;1:7. doi: 10.1186/2047-0525-1-7 (PMC3964337; doi:10.1186/2047-0525-1-7)
Supplement: Additional file 1 — Table S2. Percentage failure to reach target blood glucose levels. [file 2047-0525-1-7-S1.doc]

**Table 2: % Failure to reach target Blood Glucose levels.**
